# Frequency and associated factors of amnestic mild cognitive impairment at four senior citizen clubs in Lima, Peru

**DOI:** 10.1590/1980-57642018dn13-030009

**Published:** 2019

**Authors:** Sofía S. Sánchez, Jesus Abanto, Arantxa Sanchez-Boluarte, Alicia Boluarte-Carbajal, Danilo Sanchez-Coronel, Nilton Custodio-Capuñay, Frine Samalvides-Cuba

**Affiliations:** 1 Universidad Peruana Cayetano Heredia Faculty of Medicine Lima Peru Faculty of Medicine, Universidad Peruana Cayetano Heredia, Lima, Peru.; 2 Instituto Nacional de Ciencias Neurológicas Centro Básico de Investigación en Examenes Auxiliares en Parasitosis del Sistema Nervioso Lima Perú Centro Básico de Investigación en Examenes Auxiliares en Parasitosis del Sistema Nervioso, Instituto Nacional de Ciencias Neurológicas, Lima, Perú.; 3 Universidad Cesar Vallejo Lima Peru Universidad Cesar Vallejo, Lima, Peru.; 4 Universidad Norbert Wiener Lima Peru Universidad Norbert Wiener, Lima, Peru.; 5 Instituto Nacional de Ciencias Neurológicas Departamento de investigación docencia y atención especializada en enfermedades neurodegenerativas Lima Peru Departamento de investigación docencia y atención especializada en enfermedades neurodegenerativas, Instituto Nacional de Ciencias Neurológicas, Lima, Peru.; 6 Instituto Peruano de Neurociencias Unidad de investigación Lima Perú Unidad de investigación, Instituto Peruano de Neurociencias, Lima, Perú.; 7 Instituto Peruano de Neurociencias Servicio de neurología Lima Perú Servicio de neurología, Instituto Peruano de Neurociencias, Lima, Perú.; 8 Hospital Cayetano Heredia Lima Peru Hospital Cayetano Heredia, Lima, Peru.

**Keywords:** mild cognitive impairment, memory, alteration test, dementia, comprometimento cognitivo leve, memória, teste de alteração, demência

## Abstract

Amnestic mild cognitive impairment (aMCI) is a subtle alteration in cognitive function that does not affect day-to-day activities and can precede Alzheimer's dementia. An increase in the prevalence of both these conditions is expected given the growing elderly population and recognizing risk factors can help reduce the burden.

**Objective:**

the aim of this study was to determine the frequency and associated factors of aMCI in senior citizen clubs (SCC) at four districts with different socioeconomic status in Lima, Peru.

**Methods:**

we applied Petersen's criteria to determine the presence of the condition in an interview which included the use of the Memory Alteration Test (M@T) and the Pfeffer Functional Activity Questionnaire (PFAQ).

**Results:**

sixty-three out of 352 (17.9%) participants had aMCI. Factors associated with this condition were older age, fewer years of education at marriage whereas being from the SCC La Molina (district with highest socioeconomic status and resources for activities for the elderly) were associated with not having aMCI. There was no difference for sex, body mass index or history of hypertension.

**Conclusion:**

this predementia stage is frequent and usually undetected in urban Lima. Tools such as the M@T could help general practitioners detect this condition before its progression to dementia.

## RESUMO

O comprometimento cognitivo leve amnésico (aMCI) é uma alteração sutil na função cognitiva que não altera as atividades do dia-a-dia e pode preceder a demência de Alzheimer. Um aumento na prevalência de ambas as condições é esperado devido ao crescimento da população idosa e o reconhecimento de fatores de risco pode ajudar a reduzir a carga de doença.

**Objetivo:**

o objetivo deste estudo foi o de determinar a freqüência e fatores associados a MCI em clubes de cidadãos idosos (CCI) em quatro distritos com diferentes condições socioeconômicas em Lima, Peru.

**Métodos:**

aplicamos os critérios de Petersen para determinar a presença da condição em uma entrevista que incluiu o uso do Teste de Alteração de Memória (M@T) e do Questionário de Atividades Funcionais de Pfeffer (QAFP).

**Resultados:**

sessenta e três dos 352 (17,9%) participantes tinham MCI. Os fatores associados a essa condição foram idade avançada, menor tempo de estudo ao se casar enquanto ser provenientes do CCI La Molina (distrito com maior nível socioeconômico e recursos para atividades para idosos) foram associados a não possuir um MCI. Não houve diferença por sexo, índice de massa corporal e história de hipertensão.

**Conclusão:**

este estágio de pré-demência é frequente e geralmente não detectado na cidade de Lima. Ferramentas como o M@T poderiam ajudar os clínicos gerais a detectar essa condição antes da progressão para a demência.

Neurodegenerative diseases, including dementia, primarily affect elderly people, a steadily growing strata of the population.^1^ In Peru, one in ten people were older than 60 years old in 2015 and it is estimated that this number will increase to 13% of the total population of Peru by 2025.^2^^,^^3^Amnestic mild cognitive impairment (aMCI) is recognized as a condition that precedes Alzheimer's disease (AD), the most common type of dementia. aMCI is characterized by a cognitive alteration so subtle that it does not alter the patient's functioning, and can be overlooked in medical care.^4^^,^^5^ AD severely affects the patient's cognitive functions and quality of life, and has a high social and economic impact.^1^^,^^6^ It is also estimated that 10% of patients with aMCI will progress to dementia every year.^7^^-^9

The prevalence of this condition ranges from 1% (Canada) to 28.3% (New York, USA) in older adults.^10^ Studies from Latin America (Colombia, Argentina, Mexico) have reported frequencies of 9.1-10.8%.^11^^-^13 By contrast, in Peru, the reported crude prevalence of aMCI was 3.1%.^14^ However, given that the prevalence of dementia in a Peruvian urban population is 6.5%, the prevalence of this prior stage is expected to be higher.^15^ The heterogeneity of these results in similar studies is probably due to the use of different parameters to define aMCI criteria. For example, these studies used different methods to objectively assess this condition, ranging from the use of different neuropsychological questionnaires to an extensive evaluation by a multidisciplinary team.

One of the tools to evaluate aMCI is the Memory Alteration Test (M@T). This is a brief neuropsychological verbal test that does not require extensive training and can be applied by primary care professionals.^16^^,^^17^ This test has been validated in Peru for adults with a minimum of 6 years of education, and has shown high sensitivity (98.3%) and specificity (97.8%) for the detection of aMCI, surpassing the commonly used Mini-Mental State Examination (MMSE; sensitivity: 83.89%; specificity: 68.89%).^18^ The availability of sensitive and easy-to-use tests like the M@T could provide general practitioners with an accessible tool in scenarios where the lack of specialized professionals and resources prevent the treatment of the elderly population at risk.^16^

Known risk factors for this condition are age, history of stroke, and level of education.^19^^,^^20^ Level of education is highly related to socioeconomic status, which has also shown an association with aMCI.^21^ Cardiovascular risk factors (CVF), such as arterial hypertension, type 2 diabetes mellitus, obesity, hypercholesterolemia and tobacco smoking, have also shown a positive association.^20^ Variation in risk factors according to sex has been observed. In women, the risk factors which contributed most were having a lower level of education and not attending meetings of a religious nature. In males, these factors were older age and a history of stroke.^11^^,^^19^^,^^22^^-^24 Lifestyles play an important role in the development of this condition. The protective factors identified were regular physical activity, a healthy diet, higher education and routinely performing mental activities, like reading, resolving puzzles (crossword, Sudoku, etc.) or frequently using the computer.^19^^,^^20^ In our milieu, risk factors for aMCI have not yet been described.

Given the growing proportion of population at risk of dementia in Peru, it is important to describe this ‘pre-dementia' stage in an effort to recognize modifiable factors and implement effective intervention in the near future. aMCI early detection and the delay of progression to AD could have a crucial impact on the well-being of patient and their family members, as well as on the Peruvian economy. The aim of this study is to determine the frequency of aMCI in the elderly population from four senior citizen clubs (SCC) in Lima, Peru using the M@T and to explore factors (age, sex, marital status, years of education, body mass index (BMI), history of hypertension and district of residence) associated with this condition.

## METHODS

### Participant selection

This is a cross-sectional study that included adults older than 60 years old, with complete basic education (6 years of schooling), attending SCC from 4 districts in Lima, Peru (La Molina, Jesus Maria, Carabayllo and Cercado de Lima). The clubs were chosen according to their socioeconomic differences between their respective locations^25^ and the accessibility they provided. The sample size was 71 subjects per SCC and was calculated based on the approximately 2500 subscribed members in each SCC (1000 in total), for an estimated prevalence of 5%, based on a previous study in Peru that reported 3.1%,^11^^,^^14^ and a confidence interval (CI) of 95% with an estimated loss of 5%. The enrolment period was 1 week per SCC and the sampling was by convenience. People interested in participating were interviewed for more information before participation (See *Ethics* section). Five interviewers (the first three authors and two psychology students) were trained by experts to apply the test with similar consistency and to explain the informed consent form to the participants. Participants that failed to complete all the interview or had a condition limiting test application (overt cognitive impairment, language or hearing disability that impeded the application/completion of the tests, or previously confirmed dementia diagnosis) were not included.

### Senior Citizen Clubs (SCC)

The Peruvian Ministry of Women and Social Development implemented *Centros Integrales del Adulto Mayor* (translated as Senior Citizen Clubs, SCC) all over Peru.^26^ These centers are dedicated to organizing recreational and health promoting activities for the local elderly citizens. Although the same goal is shared across centers, different activities and resources are used according to the district income. The investment in each SCC activities and resources differ according to socioeconomic status of the district. We selected 4 SCC from Carabayllo, Cercado de Lima, Jesus Maria and La Molina districts (from the lowest to the highest socioeconomic status of the four).^2^

### aMCI criteria

Once enrolled, the subjects had to meet to following criteria (Petersen's criteria) in order to establish aMCI status:^10^

(i) Subjective loss of cognitive function: reported as cognitive complaints in the last month (memory loss). This was reported by the subject during the interview.
(ii) Preserved functioning. We applied the Pfeffer Functional Activities Questionnaire (PFAQ) to those participants who reported cognitive complaints and had preserved functioning .
(iii) Objectively measured cognitive impairment. We applied the M@T to those participants who reported cognitive complaints and met the previous two criteria.
(iv) Non-dementia. Reported by the patient and caregiver and corroborated by the results of the M@T. Patients with a M@T score below 28 were excluded from analysis for marked cognitive impairment compatible with dementia and were referred to a neurologist.

**Memory Alteration Test (M@T) -** Appendix 1 shown at: http://www.demneuropsy.com.br/imageBank/pdf/Artigo-09-(Appendix-01-02).pdf This test contains 40 questions with a maximum score of 50. It evaluates 5 areas: encoding (5 points), orientation (10 points), semantic (15 points), free recall (10 points), and cued recall (10 points). If the total score is <28, 28-36 or >36, the subject is classified as having dementia according to the M@T, having MCI (aMCI+) or having no amnestic cognitive impairment according to the M@T (aMCI-), respectively. Patients that were classified as having dementia according to the M@T were not included in the analysis and were referred to a neurologist for further studies. Interviewers were trained before applying this test.^15^

**Pfeffer Functional Activity Questionnaire (PFAQ) -** Appendix 2 shown at: http://www.demneuropsy.com.br/imageBank/pdf/Artigo-09-(Appendix-01-02).pdf

The PFAQ consists of 10 items, each scored from 0 (independent) to 3 (fully dependent). A total score of 9 or more is classified as impairment in functioning. Interviewers were trained before applying the test.

### Associated factors

During the interview, sociodemographic data were collected, including age, sex, marital status, years of education, district of the SCC. Any previous history of hypertension was also registered. In order to calculate BMI, we measured height (H, in meters) and weight (W, in kilograms) were measured and the formula W divided by H squared (units: kg/m^2^).

### Statistical analysis

The data collected was transferred to a database in Microsoft Excel 2013. The sample size was calculated with the open source program *Open-epi.* Descriptive statistics were used to determine frequencies of the condition and characteristics of the population. Means and median with standard deviations and interquartile ranges were reported. Bivariate (Chi-squared and *t-*test) and multivariate analysis (logistic regression) were used to explore associated factors using the statistical software STATA 15.

### Ethics

This study was reviewed and approved by the Institutional Review Board (IRB) of the Universidad Peruana Cayetano Heredia (UPCH), Lima - Peru, in coordination with the municipalities of Jesus Maria, Cercado de Lima, La Molina, and Carabayllo districts in Lima, Peru. All participants gave their written informed consent before participation (See Appendix). This document was also reviewed and approved by the same IRB that approved the protocol.

## RESULTS

Twenty-six of the 378 participants were excluded for scoring below 28 on the M@T (see aMCI criteria in methods section) or for failing to complete all the interview. The mean age of the remaining 352 participants was 70.91±7.07 years. Three quarters (82.9%) were women. More than half of all participants (51.3%) were married. About one in ten (11.9%, n=42) were older than 80 years. Two thirds (63.1% n=214) had a BMI greater than 25 kg/m^2^ and 33.2% (n=116) reported a history of arterial hypertension. In the overall group, participants had similar proportions of educational level, although this was slightly higher in the group with 11-15 years of education (Table 1). The mean number of years of education was 11.9±3.7 years. Of all participants, 32.1% (n=113) belonged to the SCC La Molina; 24.2% (n=85) Jesus Maria; 23.6% (n=83) Carabayllo and 20.2% (n=71) to the Cercado de Lima. Participants from the SCC Carabayllo had the lowest mean years of education (9.42±3.44 years) while La Molina had the highest (13.08±3.17 years).

**Table 1:** Sociodemographic characteristics of participants (n=352).

Characteristic		Total n (%)
Age (years)*		70.91±7.07
Age group	60-64	69 (19.6)
	65-69	98 (27.8)
	70-74	77 (21.9)
	75-79	65 (18.5)
	≥80	42 (11.9)
Sex	Female	292 (82.9)
	Male	60 (17.1)
Marital status	Single	51 (14.5)
	Married	180 (51.3)
	Widowed	93 (26.5)
	Divorced	27 (7.7)
Educational level	6-10	91 (25.8)
	11-15	152 (43.2)
	>16	109 (31.0)
History of arterial hypertension		116 (33.2)
BMI	< 25	125 (36.9)
	≥25	214 (63.1)
aMCI	Yes	63 (17.9)

* mean±SD

Participants from SCC La Molina and Jesus Maria had more years of education than the individuals from the other two clubs. Also, there were more people older than 80 years of age in the aforementioned SCC. Age, sex, marital status and BMI of participants showed no statistically significant difference between different SCC locations (p>0.05, see Table 4).

**Table 2:** Baseline sociodemographic characteristics of sample according to presence/absence of aMCI (n=352).

Characteristics		aMCI+ (n=63*)	aMCI- (n=289*)	p-value
Age (years)*		75.21±7.20	69.99±6.70	<0.001^a^
Age group	60-64	2 (2.9)	67 (97.1)	<0.001^b^
	65-69	14 (14.3)	84 (85.7)	
	70-74	11 (14.3)	66 (85.7)	
	75-79	19 (29.2)	46 (70.8)	
	≥80	16 (38.1)	26 (61.9)	
Sex	Female	52 (82.5)	240 (83.0)	0.923^c^
	Male	11 (17.5)	49 (17.0)	
Marital status	Single	11 (21.6)	40 (78.4)	0.339^b^
	Married	28 (15.6)	152 (84.4)	
	Widowed	21 (22.6)	72 (77.4)	
	Divorced	3 (11.1)	24 (88.9)	
Years of education		11 (6-11)	13 (11-16)	<0.001^d^
Educational level	6-10	31 (49.2)	60 (20.8)	
	11-15	23 (36.5)	129 (44.6)	<0.001^c^
	>16	9 (14.3)	100 (34.6)	
History of arterial hypertension	Yes	28 (44.4)	88 (30.4)	0.037^c^
	No	35 (55.6)	198 (68.5)	
Body mass index	<25	34 (53.9)	180 (62.3)	0.187^c^
	≥25	27 (42.9)	98 (33.9)	
District of origin	Jesús María	14 (16.5)	71 (83.5)	0.002^c^
	La Molina	9 (8.1)	104 (92.0)	
	Carabayllo	21 (25.3)	62 (74.7)	
	Cercado de Lima	19 (26.8)	52 (73.2)	

Participants with missing values for some characteristics were not included in analysis.

a t-test;

b Fisher;

c Chi-squared;

d Mann-Whitney tests were used for bivariate analysis because the variable did not have a normal distribution.

**Table 3:** Factors associated with presence of aMCI, determined by bivariate analysis and multiple regression (n=352).

Variables		Bivariate analysis				Multiple regression		
		OR	95% CI	P		PR	95% CI	p
Sex	Female	Ref.				Ref.		
	Male	1.03	0.57-1.86	0.923		0.73	0.39-1.34	0.311
Age	60-64	Ref.						
	65-69	4.93	1.15-21.04	0.031		5.64	1.38-22.96	0.016
	70-74	4.93	1.13-21.51	0.034		4.71	1.08-20.56	0.039
	75-79	10.08	2.44-41.68	0.001		9.23	2.25-37.91	0.002
	≥80	13.14	3.17-54.4	0.000		15.33	3.79-61.99	0.000
Years of education	6-10	Ref.				Ref.		
	11-15	0.44	0.27-0.71	0.001		0.56	0.34-0.93	0.024
	>16	0.24	0.12-0.48	0.000		0.32	0.15-0.66	0.002
SCC location	Jesús María	Ref.				Ref.		
	La Molina	0.48	0.22-1.07	0.071		0.52	0.25-1.08	0.080
	Carabayllo	1.54	0.84-2.82	0.165		1.20	0.65-2.22	0.560
	Cercado de Lima	1.62	0.88-3.01	0.122		1.46	0.80-2.67	0.216

*Adjusted for sex, years of education and SCC location.

**Table 4:** Characteristics of study participants by SCC location.

Characteristics		SCC Jesús María n=85 (24.2%)	SCC La Molina n=113 (32.1%)	SCC Carabayllo n=83 (23.6%)	SCC Cercado de Lima n=71 (20.2)	p
Age (years)*		71.78 ± 7.30	70.89 ± 7.13	69.98 ± 7.58	70.99 ± 5.99	
Age group	60-64	12 (14.1)	20 (17.7)	22 (26.8)	15 (21.1)	<0.000
	65-69	26 (30.6)	35 (30.9)	24 (29.3)	13 (18.3)	
	70-74	20 (23.5)	27 (23.9)	13 (15.9)	17 (23.9)	
	75-79	13 (15.3)	15 (13.3)	14 (17.1)	23 (32.4)	
	≥80	14 (16.5)	16 (14.2)	9 (10.9)	3 (4.2)	
Sex	Female	68 (80.0)	98 (86.7)	65 (78.3)	61 (85.9)	0.923
Marital status	Single	38 (45.2)	54 (47.8)	58 (69.9)	30 (42.3)	0.341
	Married	18 (21.4)	7 (6.2)	12 (14.5)	14 (19.7)	
	Widowed	22 (26.2)	41 (36.3)	11 (13.3)	19 (26.8)	
	Divorced	6 (7.1)	11 (9.7)	2 (2.4)	8 (11.3)	
Years of education		14 (11-16)	15 (11-16)	9 (6-11)	11 (7-14)	
Educational level	6-10	9 (10.6)	11 (9.7)	45 (54.2)	26 (36.6)	<0.000
	11-15	42 (49.4)	52 (46.0)	30 (36.1)	28 (39.4)	
	>16	34 (40.0)	50 (44.3)	8 (9.6)	17 (23.9)	
History of hypertension		35 (41.7)	25 (22.3)	26 (31.7)	30 (42.3)	0.037
Nutritional status	<25	26 (30.9)	46 (45.1)	28 (33.7)	25 (35.7)	0.187
	≥25	58 (69.1)	56 (54.9)	55 (66.3)	45 (64.3)	
aMCI	Yes	14 (16.5)	9 (7.9)	21 (25.3)	19 (26.8)	0.002

We found that 17.9% (n=63) participants had aMCI. The mean age in this group was higher than in the aMCI-group (75.2 vs. 69.9 years old, respectively, p<0.001). No difference was found in aMCI frequency between males and females. Almost four out of five were women in both aMCI and aMCI- groups while in males the proportion was around 17% in both groups (p=0.923, see Table 2). Regarding marital status, 15.6% (n=28) of those with the condition were married, while in the group without cognitive impairment, the percentage of married couples was 84.4% (n=152) of the conglomerate of groups that included single, widowed and divorced subjects (p=0.02, OR 0.55, CI 0.30-0.98). The mean years of education was higher in the aMCI-group (13 vs 11 years, p<0.001). In the aMCI group, when divided by subgroup, 49.2% (n=31) had 6 to 10 years of education (lowest level of education in our study population) and the proportion of individuals with more than 16 years of education was 14.3% (n=9). In the aMCI-group, 44.6% of participants had 11 to 15 years of education (n=129), closely followed by participants with more than 16 years of education, representing 34.6% (n=100). Differences between these subgroups were statistically significant (p<0.001). There was no significant difference in the presence of aMCI for the BMI index. In terms of location, the group with the lowest frequency of aMCI was from the district of La Molina with 8.1% (n=9), followed by Jesús María with 16.5% (n=14). The districts with the highest frequency of aMCI were Carabayllo with 25.3% (n=21) and Cercado de Lima with 26.8% (n=19) (p=0.002, see Table 2).

Being older was associated with the condition. On bivariate analysis, aMCI was 10 times more frequent in participants aged over 75 years (p=0.001, OR 10.08, CI 2.44 - 41.68). Also, having more than 16 years of education was associated with not having the condition (p<0.001, OR 0.24, CI 0.12-0.18). Belonging to the SCC from La Molina district was associated with a lower frequency of aMCI, but was not statistically significant (p=0.071, OR=0.48, CI=0.22-1.07, see Table 3). Multivariate analysis showed similar results (See Table 3). All analyses were adjusted for sex, years of education and SCC location.

## DISCUSSION

The frequency of aMCI in this study was 17.9%, similar to the prevalence found in Tremembé, Brazil.^27^ In other Latin American countries, a prevalence of around 10% was reported.^2^^-^4 A multicenter study that included Peru reported a prevalence of 3.1%, low compared to other countries such as the United States, Italy and Spain (15.7%, 18.2%, 18.5%, respectively).^5^^-^28 This variability is probably due to the lack of homogeneity of the criteria that defined this entity, the type of sampling and the neuropsychological tests used.^9^^-^12^,^^29^^,^^30^ In addition, the lack of epidemiological studies in our region and the potential underdiagnosis of this entity should be considered.

Participants who presented aMCI had an average age greater than those without impairment, which is in agreement with previous literature. This condition was found in 10% of individuals aged 70-79 years and 25% in those aged 80-89 years.^5^^,^^11^^,^^13^^-^15 The subgroup of those over 75 years old had a ten times higher risk of having this condition. Considering that this subgroup is at greater risk of developing AD, this finding highlights the importance of screening for aMCI in older adults, acting on risk factors, delaying both onset and progression of dementia and taking family and social health measures related to dementia care.^6^^,^^12^^,^^16^

The other associated factor was educational level. Higher-educated elderly (>11 years of education) had half the risk of having aMCI compared to those with basic education (6-10 years of education). This finding agrees with several studies, which reported that a higher level of education is a protective factor for cognitive impairment, as well as for the subsequent development of dementia.^15^^,^^17^^,^^31^^-^35 This reinforces the idea that intellectual activity delays the degeneration of cognitive function. There is evidence that a higher cognitive reserve, which refers to differences between individuals that will be more resistant to brain changes, provides more tolerance to neurodegenerative disease. Intellectual and physical activity at all stages of ageing, even in later life, can increase cognitive reserve and may be the reason for the delay in progression of dementia.^36^

Also, being married was associated with less deterioration, corroborating previous reports. This can be attributed to the fact that married people, when not alone, have greater social activities than those of other marital status and are less predisposed to develop depression. However, factors such as years of marriage and quality of interaction between the couple were not studied. Double the risk in single people has been reported compared to married couples at midlife and a strong sociogenetic role could play a role in the development of AD.^37^ Further studies are suggested to evaluate their possible role as a protective factor.^10^^-^12 Attending the SCC La Molina was also a factor associated with not having the condition, even after adjusting for years of education. This may be due to the previously discussed socioeconomic differences between districts. In addition, this SCC had better infrastructure, human resources that included psychologists, and intellectual activities such as reading clubs, artistic activities, among others, which were also run on a more regular basis. Inequality among districts should be highlighted, this time related to access to public services of higher quality for the elderly, limited by the socioeconomic development of the SCC location. Factors related to the interaction of the elderly for the center such as years of membership and regularity of attendance were not evaluated because of lack of reliable records. One of the strengths of this study is the multicenter approach that included participants from different socioeconomic backgrounds. These centers are potential places for intervention where the public district offices can participate. Another strength of the present study was the use of the M@T. This instrument is brief and easy to apply, designed as a screening test for use by the professionals of the primary care centers and that has shown high diagnostic performance compared to the MMSE.^16^ We recognize that secondary causes of aMCI, such as B12 deficiency, hypothyroidism, depression and drug use, were not determined. Also, we relied solely on the M@T ,which serves more like a tool for initial screening than for establishing a conclusive diagnosis of this condition. It is also important to point out that the majority of participants in this study were female, at a ratio of 4:1 compared to male participants. Several studies show this disproportion, but not to this magnitude, therefore caution should be exercised when attempting to extrapolate the study findings to males.

We conclude that the frequency of this entity in the geriatric population of the SCC was 17.9%. Associated factors were age, history of hypertension, and years of education. Risk factors should be identified and screening tests carried out among elderly in primary care to establish an early diagnosis and implement interventions in this predementia stage, thereby slowing or preventing transition to AD.
